# Triplet chemotherapy combination with cisplatin, gemcitabine and docetaxel in patients with chemotherapy-naive advanced non-small cell lung cancer

**DOI:** 10.3892/ol.2013.1205

**Published:** 2013-02-21

**Authors:** FARUK TAS, FATMA SEN, NESE GUNEY, SERKAN KESKIN, HAKAN CAMLICA

**Affiliations:** Department of Medical Oncology, Institute of Oncology, Istanbul University, Istanbul 34390, Turkey

**Keywords:** cisplatin, docetaxel, gemcitabine, non-small cell lung cancer, triplet chemotherapy, weekly

## Abstract

The synergistic effects of new generation chemotherapeutics when combined with cisplatin have encouraged the development of new triplet combination regimens in the treatment of advanced non-small cell lung cancer (NSCLC). The aim of this study was to evaluate the feasibility of triplet chemotherapy using weekly cisplatin-gemcitabine-docetaxel (CGD) for patients with chemotherapy-naive NSCLC. Twenty-seven patients with stage IIIB/IV disease and performance status of 0 to 2 were included in this prospective trial. A combination of gemcitabine 750 mg/m^2^, cisplatin 25 mg/m^2^ and docetaxel 25 mg/m^2^ was administered on days 1, 8 and 15, with cycles repeated every 3 weeks. Leucopenia and/or neutropenia and to a lesser extent thrombocytopenia were the main dose-limiting toxicities. Grade III–IV neutropenia and thrombocytopenia occurred in 26 and 7% of the patients, respectively. Only one patient developed febrile neutropenia. Dose reductions were required in 26% of patients, delays in 44% of patients and early treatment discontinuation in 15% of patients. The overall response rate was 52% and all of them experienced a partial response. The median progression-free (PFS) and overall survival (OS) times were 6 and 13 months, respectively. The one-year survival rate was 46%. In conclusion, weekly administration of CGD is an active first-line therapy with acceptable toxicity in advanced NSCLC patients.

## Introduction

Non-small cell lung cancer (NSCLC) accounts for over 80% of patients with lung cancer, and nearly two-thirds of NSCLC patients present with advanced disease (stage IIIB/IV). Cisplatin-based chemotherapy with best supportive care (BSC) has been demonstrated to have statistically significant survival advantage when compared with BSC alone in advanced NSCLC patients (1,2). Cisplatin-based doublet chemotherapy with third generation cytotoxic agents, such as the taxanes vinorelbine or gemcitabine has further improved survival rates when compared with older regimens such as vindesine or etoposide (3,4). Currently, doublet chemotherapy including a platinum and a third generation cytotoxic is accepted as a standard regimen for advanced NSCLC. Adding a third active drug is one of the further efforts to improve treatment outcome.

Gemcitabine, a pyrimidine nucleoside antimetabolite, is one of the most promising new cytotoxic agents. It has shown activity in a variety of solid tumors, but appears to be very active in the treatment of NSCLC (5). Gemcitabine in combination with cisplatin has synergistic effects without overlapping toxic side effects (6). Many studies evaluated this combination with different treatment schedules and they showed response rates varying between 37 and 42% and a median overall survival (OS) period of between 8.4 and 13.5 months (6–11). A recent meta-analysis showed an absolute benefit (3.9%) in 1-year OS in favor of regimens containing gemcitabine and platinum, with a significant reduction in both overall mortality and disease progression (12). Thus, gemcitabine combined with cisplatin is currently considered to be one of the standard regimens in the treatment of advanced NSCLC (13–15).

Docetaxel, a semisynthetic taxoid, possesses significant activity as a single agent in the treatment of patients with NSCLC. A review demonstrated that the results from significant phase I and II studies showed significant antitumor activity and tolerability of docetaxel combined with platinum compounds for patients with advanced NSCLC (16). Thus, the effectiveness of docetaxel as a single agent or cisplatin-based doublet has already been accepted in treatment of advanced NSCLC (15,17,18).

The aim of this study was to investigate the feasibility and effectiveness of cisplatin-gemcitabine-docetaxel (CGD) triplet given weekly in patients with chemotherapy-naive advanced stage NSCLC.

## Materials and methods

### Patient eligibility criteria

Histologically or cytologically confirmed NSCLC patients with Eastern Cooperative Oncology Group (ECOG) performance status (PS) of 0 to 2 and advanced stage disease (IIIB/IV) were included. Patients who had brain metastases were required to have completed cranial radiotherapy at least 3 weeks prior to triplet chemotherapy with a stable neurologic status. Other inclusion criteria were adequate functioning of bone marrow (white blood cell count ≥3,000/mm^3^, hemoglobin ≥9 g/dl, platelets ≥100,000/mm^3^), liver (total bilirubin level ≤1.5 mg/dl, alanine and aspartate transaminase levels less than twice the upper limit of normal) and kidney (serum creatinine level <1.5 mg/dl). Patients with a previous history of chemotherapy for advanced stage NSCLC, and major comorbidities such as severe cardiopulmonary dysfunction, recent history of myocardial ischemia, uncontrolled arrhythmia and active infection were excluded. All patients gave written informed consent before treatment and the study was approved by the local institutional scientific committee.

### Baseline evaluation

Pretreatment assessment routinely included medical history, physical examination, complete blood count, serum biochemistry, electrocardiogram (ECG), chest computed tomography (CT), abdominal CT and/or magnetic resonance imaging (MRI), bone scan or positron emission tomography (PET/CT) and cranial MRI when indicated. Tumor stages were assessed according to the TNM classification of International Union Against Cancer (19).

### Treatment schedule

Gemcitabine was administered intravenously (i.v.) as a 30 min infusion with saline at a dose of 750 mg/m^2^, cisplatin and docetaxel were given i.v. as a 1 h infusion with saline at a dose of 25 mg/m^2^ on days 1, 8, and 15. Treatment cycles were repeated every 3 weeks on an outpatient basis unless disease progression or severe toxicity was experienced. Premedication consisted of antiemetic combination with standard serotonin antagonists and dexamethasone. Granulocyte colony stimulating factor (G-CSF) was not used prophylactically during the first cycle of the study, but it was recommended for patients who had previously experienced either febrile neutropenia or grade IV neutropenia lasting more than 5 days. All patients who developed febrile neutropenia were also eligible for prophylactic growth factor administration in the next cycles.

### Toxicity and dose delay or modifications

Complete blood counts were performed on a weekly basis. Serum biochemistry and physical examination including the determination of ECOG PS and vital signs were performed one day before each chemotherapy cycle. Common toxicity criteria of NCI (CTCv3.0) had been used. The doses of chemotherapeutic agents were modified for hematological and severe nonhematological toxicities except for emesis. Treatment was delayed for one week if there was not full hematological recovery from the prior cycle of treatment. Patients requiring more than two dose reductions or a delay of more than 3 weeks were removed from the study. Patients with progressive disease at any time were withdrawn from the study.

### Response evaluation

Before each cycle, common toxicity criteria, PS and measurement of clinically assessable disease were documented. Patients were evaluated for response if they received one or more cycles of treatment. If there was no sign or symptom of progression after one cycle, response was assessed at the end of second chemotherapy cycle. Tumor response was evaluated by physical examination, imaging radiograms such as CT, MR, US and/or PET/CT. Patients with stable disease or partial response after the second cycle continued to a maximum of 4 cycles unless intolerance developed. Two additional cycles of chemotherapy were given to responsive patients. Patients with progression or severe toxicity after sufficient dose reduction discontinued the treatment.

### Statistical analyses

SPSS software (SPSS version 16, Chicago, IL, USA) was used for statistical analyses. Quantitative analyses were summarized by mean, standard error, median, minimum and maximum, and qualitative analyses were presented as frequencies and percentages. Survival analyses were estimated by the Kaplan-Meier method. P≤0.05 was considered to indicate a statistically significant result. The overall response rate in previous studies with the same triplet GCD showed a success rate of 34% (20). New patients were included in the study until a 95% confidence interval rate was reached. New patient admission was stopped after the first patient had a one year follow-up. Sample size was determined using Simon’s tables.

OS was determined as the time elapsed between the time of histological diagnosis and the date of mortality or the date of the last follow-up visit or the date of the study written, if the patient was still alive at this time. Progression-free survival (PFS) was recorded from the day of histological diagnosis to the date of first documented progressive disease or the date of mortality, regardless of its course, or to date of point if no progressive disease and no mortality appeared at this time.

## Results

### Patients

In total, 27 patients (26 males and 1 female) were treated with triplet CGD regimen between January 2008 and December 2009. The clinical characteristics of the patients are listed in Table I. The median age of patients was 59 years (range, 38–72 years). The majority were male and had PS 0–1 and stage IV disease.

### Safety

The toxicity profiles of all 27 patients are shown in Table II. Hematological toxicity was found to be the principal dose-limiting toxicity. Severe, grade III/IV neutropenia and leucopenia were observed in 26 and 19% of the patients, respectively. One patient developed febrile neutropenia with an underlying empyema and pneumothorax. Two patients required G-CSF usage due to febrile neutropenia and grade IV neutropenia without fever. Only 2 patients (7%) developed grade III thrombocytopenia. None of the patients had grade III/IV anemia. However, due to symptomatic grade II anemia, 7 patients (26%) received a total of 12 units of packed red blood cells.

Patients were treated with various antiemetics containing seratonine antagonists. Several other severe non-hematological side effects were managed according to the standard protocols. No toxic or early mortality was observed.

### Dose delivery

The patients received a total of 85 cycles of CGD with a median of 4 cycles per patient (range, 1–6). Permanent 25% dose reductions were necessary in 7 (26%) patients due to severe hematological and non-hematological toxicity. Chemotherapy administration was also delayed in 12 (44%) patients due to delayed hematological or non-hematological toxicity recovery. Treatment was discontinued in 4 (15%) patients due to severe fatigue and deteriorating performance status (2 patients) or disease progression (2 patients) after 1–3 cycles of chemotherapy.

### Efficacy

All 27 patients were evaluated for response (Table III). The overall response rate for all patients was 52% and all of them had partial response. Two patients (7%) had stable disease and the remaining 11 patients (41%) showed progression under treatment.

### Survival analyses

**Figure 1:**
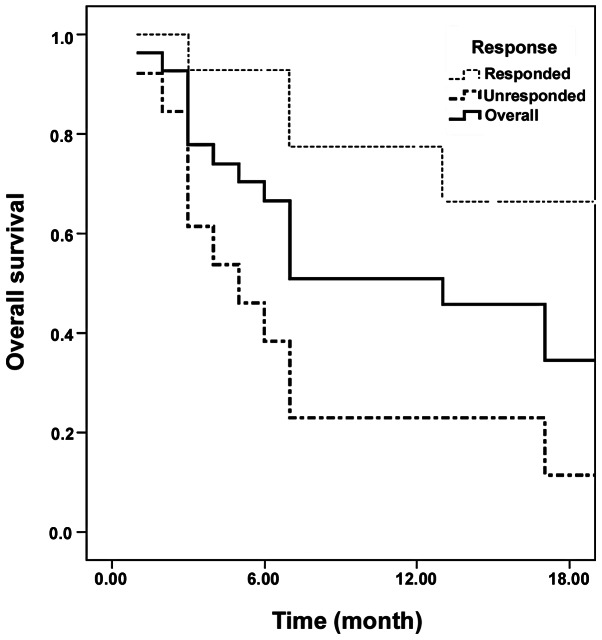
Overall survival (OS) curves.

The median follow-up of the patients was 7 months (range, 2–18). At the time of last follow-up, 10 (37%) patients were alive. The median PFS was 6 months (95% CI, 3–9). The median OS was 13 months (95% CI, 6–20) (Fig. 1). The 1-year OS rate was 46% (range, 2–30 months). The median PFS of responders to chemotherapy was 11 months (95% CI, 9–13) and the median PFS of non-responders was 3 months (95% CI, 1–5) (P=0.02). The median OS of responders to chemotherapy could not be reached, as sufficient mortality was not observed; however, the median OS of non-responders was 5 months (95% CI, 2–9) (P=0.01; Fig. 1).

## Discussion

Based on the results of several randomized trials, there is sufficient evidence to conclude that adding a drug to a single agent or to a two-agent regimen increased the tumor response rate in patients with advanced NSCLC, although its impact on survival remains controversial (21,22).

A recent meta-analysis showed that adding an agent to doublet therapy did not provide a better survival than that of doublet chemotherapy (23). Additionally, toxicity was increased significantly. Most of the studies, including that meta-analysis, combined first or second generation agents. It was suggested that the third generation agent was diluted and had less effect. A review of the studies performed with triplet cytotoxic chemotherapy in advanced NSCLC demonstrated that triplet therapy with third generation agents increased response rates of tumors at the expense of increased toxicity (24). Although triplet chemotherapy had a better OS compared with doublet therapy, it was not statistically significant.

Phase I trials determined that 75 mg/m^2^ docetaxel and 75 mg/m^2^ cisplatin is the recommended dose for phase II and III trials (16). Overall, response rates with docetaxel and cisplatin have ranged from 21 to 48%. Median survival of 8 to 13 months has been achieved in phase II trials. A recent phase I/II trial investigated weekly consecutive administration of docetaxel at a dose of 40 mg/m^2^ on days 1, 8 and 15 for 3 weeks plus cisplatin at a dose of 75 mg/m^2^ on day 1 every 4 weeks (25). It was found to be tolerable and effective (objective response rate of 27.7%) with minimal myelosuppression in chemotherapy-naive patients with advanced NSCLC (26).

A phase I/II study with cisplatin, gemcitabine and docetaxel in patients with advanced NSCLC has been conducted (20). All drugs (cisplatin 40 mg/m^2^, docetaxel 30 mg/m^2^ and gemcitabine 800 mg/m^2^) were given on days 1 and 8, repeated every 3 weeks. The objective response rate was found to be 34% with tolerable toxicity. As the possibility of obtaining better response and survival gain, but same levels of toxicity was shown when comparing with cisplatin-based doublets, we conducted the current study with this triplet combination of CGD with weekly schema in order to lower side effects.

This is the first clinical study of scheduled weekly continuous administration evaluating the efficacy of this combination in this setting. The current study demonstrated that the combination of two new generation drugs, gemcitabine and docetaxel, with cisplatin and weekly continuous administration of this triplet, has favorable antitumor activity in patients with chemotherapy-naive advanced NSCLC. When compared with cisplatin-based doublet regimens with either gemcitabine or docetaxel, concurrent delivery of both drugs with cisplatin weekly appears to enhance antineoplastic activity, as shown b ∼50% higher response rate in this study than in those previously reported (20).

This weekly triplet regimen in the present study was also found to be safe and well tolerated and adverse events were mild to moderate in the majority of the patients. Compared with regimens with one week interruption, weekly continuous administration of triplet chemotherapy regimen diminishes the frequency of myelotoxicity, nausea, infection (which were the principle toxicities) or other adverse events. Myelosuppression was infrequent and readily manageable. Only one patient had febrile neutropenia and myelosuppression-related discontinuation of treatment did not occur in any patient. Moreover, there were no treatment-related mortalities. The dose and schedule of treatment used in the current study appears to be tolerable and reasonable for patients in this setting.

In conclusion, cisplatin-based triplet treatment with new generation drugs increased the response rate with acceptable side effects and improved OS or PFS. This should encourage further studies with triplet cytotoxic chemotherapy regimens at reduced doses as first line chemotherapy in advanced NSCLC patients.

## Figures and Tables

**Table I:** Characteristics of patients and tumors.

Parameter	Value
Age (years)	
Median	59
Range	38–72
Gender, n	
Female	1
Male	26
Performance status, n	
0	14
1	9
2	4
Histology, n	
Adenocarcinoma	13
Squamous cell carcinoma	11
Undifferentiated	3
Tumor stage, n	
IIIB	2
IV	25

**Table II:** Toxicity profile.

	NCI toxicity (no. of patients)			
Toxicity	I	II	III	IV
Hematological				
Leucopenia	1	10	5	0
Neutropenia	2	7	6	1
Anemia	3	14	0	0
Thrombocytopenia	3	4	2	0
Biochemical				
Creatinine	2	0	0	0
Transaminase	0	2	0	0
ALP	0	0	1	0
Bilirubin	0	0	0	0
Non-hematological				
Alopecia	5	5	0	0
Diarrhea	5	0	1	0
Constipation	3	0	0	0
Nausea	10	5	0	0
Vomiting	5	6	0	0
Pulmonary	2	4	1	2
Stomatitis	7	2	0	0
Fatigue	1	1	0	1
Insomnia	0	2	0	0
Allergic reaction	2	0	0	0
Dysphasia	1	1	0	0
Hearing loss	0	0	0	0

ALP, alkaline phosphatase; NCI, National Cancer Institute.

**Table III:** Response evaluation.

Response	No.	%	95% CI
Complete response	0	0	0–13
Partial response	14	52	32–71
Stable disease	2	7	1–24
Progression	11	41	22–61

CI, confidence interval.
